# Does being a retired or employed caregiver affect the association between behaviours in Alzheimer’s disease and caregivers’ health-related quality-of-life?

**DOI:** 10.1186/s13104-017-3099-2

**Published:** 2017-12-21

**Authors:** Melissa Majoni, Mark Oremus

**Affiliations:** 1 Department of Epidemiology and Biostatistics, Western University, 1151 Richmond Street, London, ON Canada; 2 School of Public Health and Health Systems, University of Waterloo, 200 University Avenue West, Waterloo, ON Canada

**Keywords:** Alzheimer’s disease, Behaviour, Caregiver, Employment, Health-related quality-of-life, Retirement

## Abstract

**Objective:**

We examined whether caregivers’ employment status (i.e., retired or employed) might modify the association between the behaviours of persons with Alzheimer’s disease (PwAD) and caregivers’ health-related quality-of-life (HRQoL). Data came from a cross-sectional study of the primary informal caregivers of 200 persons with mild or moderate Alzheimer’s disease. Caregivers completed the EQ-5D-3L to rate their HRQoL and generate health utility scores, and the Dementia Behaviour Disturbance Scale (DBDS) to assess the degree to which PwAD exhibited each of 28 behaviours. Caregivers’ health utility scores were regressed on overall DBDS scores, with caregiver employment status (retired, employed) treated as an effect modifier and confounder in separate regression models. We also controlled for age, sex, income, education, caregivers’ relationship to the PwAD, and whether caregivers gave up paid employment/cut down working hours to care for PwAD.

**Results:**

Effect modification by caregiver employment status is possible, with the inverse association between DBDS score and health utility score largely existing for retired versus employed caregivers. Research using larger samples and longitudinal data would further inform this area of inquiry.

## Introduction

Alzheimer’s disease (AD) is characterized by cognitive decline that disrupts the capacity to think, understand, remember and communicate information [1]. Global projections suggest more than one million cases of AD will occur by 2038 [2]. No cure exists for AD [3].

Although the clinical presentation of the disease varies, 80% of persons with AD (PwAD) can expect to exhibit multiple behavioural disturbances over time [4], including apathy, depression, aggression, anxiety, and sleep disorder [5]. These behaviours can adversely affect PwAD lifestyles and management, and caregiver health [6–8].

Caregiver health is important because PwAD receive much of their care at home and the onus of care provision generally falls on primary informal caregivers [1], typically spouses or adult children [9]. Caregiving for PwAD is a physically and emotionally challenging role, especially as the disease progresses and caregivers devote more time to meeting their loved ones’ basic needs and less time to their own interests [10]. The demands of caring for PwAD impact caregivers’ health, well-being, and health-related quality-of-life (HRQoL) [11] to the point where caregivers are called the “hidden victims” [12] or “secondary patients” [1] of AD. Evidence suggests the behavioural disturbances associated with AD have more impact on caregiver health than PwAD’s cognitive impairment [13].

Understanding the factors affecting PwAD behaviours and caregiver health is important given the crucial role of caregivers in managing AD. We undertook this study to examine whether caregiver employment status (retired versus employed full- or part-time) would modify the association between PwAD’s behavioural disturbances and caregiver HRQoL. Caregivers cover many of the costs of caring out of their own pockets. Retired caregivers who live on fixed incomes may be less able than employed caregivers to absorb these costs or seek relief through respite care or daycare programs, thereby amplifying the effects of their loved ones’ behavioural challenges on HRQoL. To date, no other study has examined the role of caregiver employment status in this area.

## Main text

### Methods

This study included 200 primary informal caregivers of persons with mild or moderate AD. These individuals were initially recruited to assess the willingness-to-pay for AD medications in a study led by one of the authors (MO) [14]. Recruitment took place in nine memory and geriatric clinics across Canada between November 2008 and August 2011. The investigators selected these clinics because they were based in research-intensive academic settings with extensive aging-related research programs, which promoted recruitment and attention to study rigour. Further details about recruitment and the study process are available elsewhere [14].

Eligible caregivers had to be retired or employed full- or part-time, be the primary informal (unpaid) caregiver, and be fluent in English or French. We excluded caregivers who reported their employment status as student, homemaker, or unemployed, who cared for someone with severe AD, or who cared for someone who could not give informed consent.

Through an in-person questionnaire, administered by trained interviewers, caregivers completed the Dementia Behaviour Disturbance Scale (DBDS) [15], which assessed the presence and intensity of 28 different PwAD behaviours on a 5-point Likert scale (0 = never, 4 = all of the time). The responses were summed to determine an overall behaviour disturbance score ranging from 0 (no disturbance) to 112 (maximum disturbance).

Caregivers also completed a generic HRQoL instrument called the EQ-5D-3L [16], which asks respondents to report their health status on each of five dimensions (mobility, self-care, usual activities, pain/discomfort, anxiety/depression). Three response options are available per dimension (no problems, some/moderate problems, extreme problems/unable to do). A Canadian algorithm converted EQ-5D-3L responses into health utility scores ranging from 0 (equivalent to death) to 1 (equivalent to perfect health) [17].

The study questionnaire also asked participants about their sex, employment status, relationship to the PwAD, type and length of care provided, satisfaction with caregiving, and level of AD knowledge [18].

For sample characteristics, the continuous variables were summarized using medians and 25th/75th percentiles and the categorical variables were summarized using frequencies. We compared sample characteristics between retired and employed caregivers using the Mann–Whitney U test for continuous variables and the Chi square or Fisher exact test for categorical variables.

We regressed caregivers’ health utility scores on DBDS scores using ordinary least squares regression and nonparametric bootstrap estimated standard errors (1000 bootstrap samples), which yielded bias corrected and accelerated confidence intervals. The initial regression model contained only DBDS score. Additional models included employment status (retired versus employed) and an interaction term (DBDS × employment status). We also constructed a full model containing DBDS score, employment status, the interaction term, and the sample characteristics that were statistically significantly different between retired and employed caregivers (Table 1).

**Table 1:** Sample characteristics (n = 200)

Characteristic	Retired (n = 140)	Employed (n = 60)	p value
Age (years)^a^	74 (68–80)	56 (51–62) n = 1 missing	< 0.0001
Dementia Behaviour Disturbance Scale score^a^	16 (10–23)	19 (10–27)	0.35
Health utility score^a^	0.80 (0.73–1.0)	0.84 (0.83–1.0)	0.02
Hours per day spent caring for PwAD^a^	2 (1–5)	2 (1–5)	0.74
Sex^b^			0.20
Male	53 (38%)	17 (28%)	
Female	87 (62%)	43 (72%)	
Education^b,c^			0.03
High school or less	52 (37%)	13 (22%)	
Technical/community college	24 (17%)	19 (32%)	
Bachelor degree	49 (35%)	18 (30%)	
Graduate degree	15 (11%)	10 (17%)	
Employment status^b^			–
Full time	0 (0%)	37 (62%)	
Part time	0 (0%)	23 (38%)	
Had to give up paid employment or reduce working hours to care for PwAD^b,d^			< 0.0001
Yes	7 (5%)	30 (50%)	
No	133 (95%)	30 (50%)	
Annual household income^b^			0.002
< $20,000	5 (4%)	0 (0%)	
$20,000 to less than $40,000	40 (29%)	11 (18%)	
$40,000 to less than $60,000	34 (24%)	8 (13%)	
$60,000 to less than $80,000	24 (17%)	8 (13%)	
$80,000 or more	27 (19%)	28 (47%)	
Missing	10 (7%)	5 (8%)	
PwAD disease severity^b^			0.33
Mild	115 (82%)	45 (75%)	
Moderate	25 (18%)	15 (25%)	
Caregiver relationship to PwAD^b^			< 0.0001
Spouse	120 (86%)	18 (30%)	
Child	8 (6%)	31 (52%)	
Other relative	8 (6%)	11 (18%)	
Friend	3 (2%)	0 (0%)	
Missing	1 (< 1%)	0 (0%)	
Length of time caregiving (year)^b^			0.59
< 1	27 (19%)	14 (23%)	
1–2	52 (37%)	26 (43%)	
3–4	25 (18%)	10 (17%)	
> 4	36 (26%)	10 (17%)	
Caregiving perceived as demanding^b^			0.52
Very demanding	26 (19%)	9 (15%)	
Somewhat demanding	81 (58%)	39 (65%)	
Not at all demanding	31 (22%)	11 (18%)	
Missing	2 (1%)	1 (2%)	
Caregiving perceived as rewarding^b^			0.32
Very rewarding	33 (24%)	17 (28%)	
Somewhat rewarding	70 (50%)	32 (53%)	
Not at all rewarding	26 (19%)	5 (8%)	
Missing	11 (8%)	6 (10%)	
PwAD living arrangements^b^			< 0.0001
Lives with caregiver	123 (88%)	28 (47%)	
Lives with someone else	3 (2%)	10 (17%)	
Lives alone	5 (4%)	14 (23%)	
Lives in an institution	8 (6%)	8 (13%)	
Missing	1 (< 1%)	0 (0%)	

^a^Median (25th–75th percentile)

^b^n (%)

^c^Completed all or some of the specified level of education

^d^Person with Alzheimer’s disease

We used multiple imputation by chained equations to account for missing values on three sample characteristics (age, relationship to PwAD, income) in the full regression model. The imputation dataset included all of the variables in the full model, with predictive mean matching to impute for age and the multinomial logit model to impute for relationship and income. The process created five imputed datasets, which were combined to obtain a new set of regression coefficients for the full model. Prior to imputation, graphical assessment showed the missing values to be missing completely at random.

We used R v3.4.1 (R Foundation for Statistical Computing, Vienna, Austria) to conduct the statistical analysis; the criterion for statistical significance was *α* = 0.05.

### Results

The sample included 140 retired caregivers, 5% of whom reported giving up paid work or reducing work hours to care for PwAD in the time prior to retirement. Many of the retired caregivers were spousal caregivers (86%), with the remaining 14% being either adult children, friends, or other relatives. Sixty caregivers reported working full-time (62%) or part-time (38%). Half of the employed caregivers gave up paid work or reduced work hours to care for their loved ones. Seventy percent of the employed caregivers were either adult children or other relatives, with the remainder being spousal caregivers (30%). Table 1 compares sample characteristics between retired and employed caregivers.

**Fig. 1:**
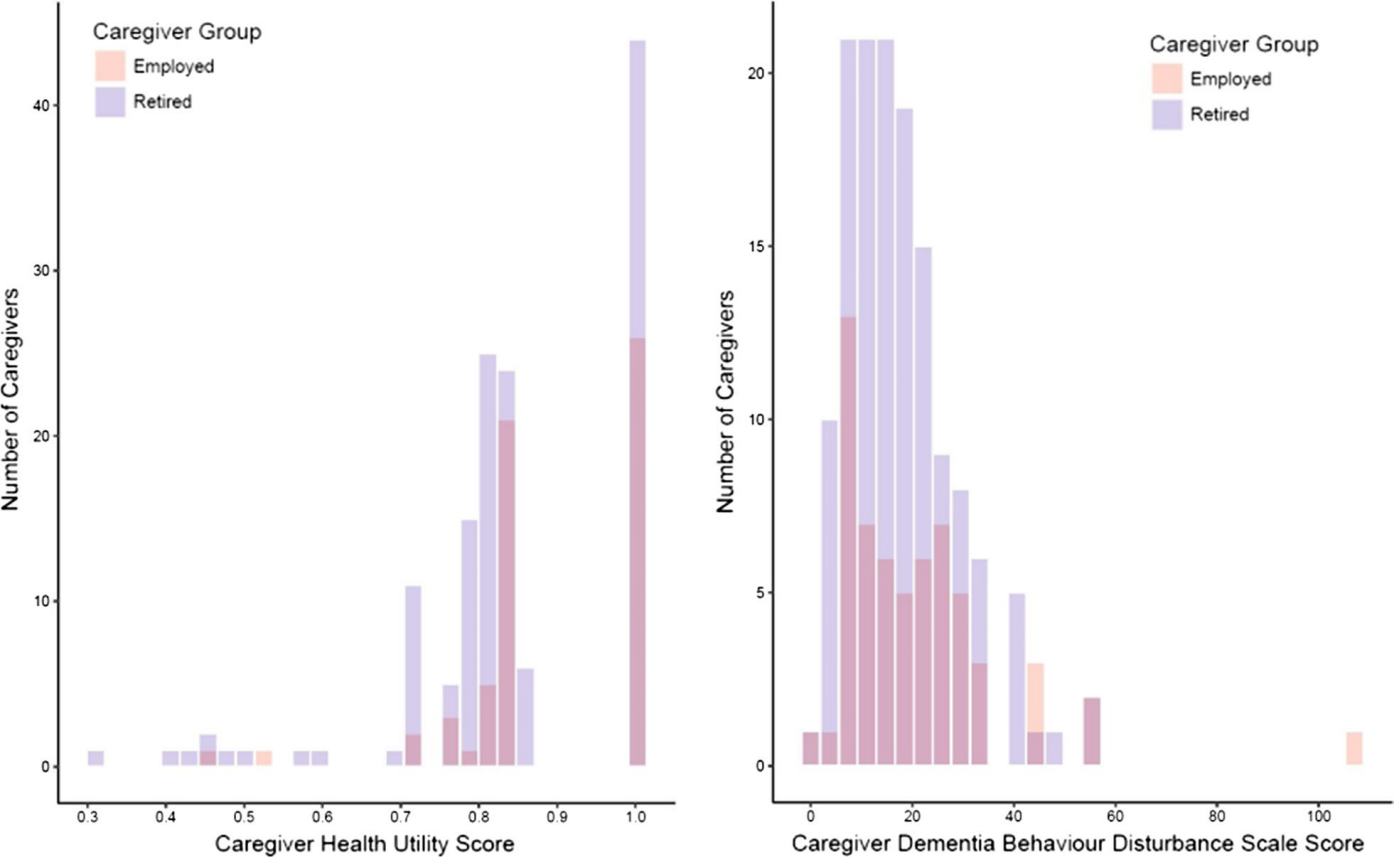
Distribution of health utility scores in retired and employed caregivers and distribution of Dementia Behaviour Disturbance Scale scores in retired and employed caregivers. Grey colour is employed caregivers overlaid on retired caregivers

Median health utility scores were 0.80 for retired caregivers and 0.84 for employed caregivers (p = 0.02); both sets of scores were left skewed (Fig. 1). Median DBDS scores were 16 for retired and 19 for employed caregivers (p = 0.35) (Fig. 1).

The interaction term for DBDS and employment status was not statistically significant in the full regression model nor the model containing only DBDS and employment status (Table 2). The interaction term’s regression coefficient was the same (i.e., − 0.003) in both models, as was the upper bound of the 95% confidence interval (CI) (i.e., 0.000).

**Table 2:** Regression analysis: caregiver health utility scores regressed on Dementia Behaviour Disturbance Scale score and caregiver employment status

	Model 1^a^	Model 2^a^	Model 3^a^	Model 4^a,b,c^
DBDS^d^	− 0.002 (− 0.004, 0.000)	− 0.001 (− 0.002, 0.001)	− 0.002 (− 0.004, 0.000)	0.000^e^ (− 0.002, 0.002)
Caregiver employment status (retired versus employed [employed = reference])	–	0.007 (− 0.057, 0.066)	− 0.05 (− 0.089, − 0.008)	0.101 (0.029, 0.189)
Interaction between DBDS and caregiver employment status	–	− 0.003 (− 0.006, 0.000)	–	− 0.003 (− 0.007, 0.000)

^a^Regression coefficient (95% bias corrected and accelerated confidence interval)

^b^Controlling for age, sex, income, education, caregivers’ relationship to the PwAD, and whether caregivers gave up paid employment/cut down working hours to care for PwAD

^c^n = 183 (other models n = 200)

^d^Dementia Behaviour Disturbance Scale

^e^− 0.0003 when expressed to four decimal places

For DBDS, the regression coefficient in all four models suggested a small inverse association between AD behaviours and caregivers’ HRQoL. The addition of covariates to the model diminished, but did not entirely eliminate, the behaviour-HRQoL association. Caregiver employment status was not a confounder because adding it to the model with DBDS alone did not change the regression coefficient for DBDS.

In the full model, the regression coefficients for DBDS and the interaction term between DBDS and employment status did not change following multiple imputation. The coefficient for employment status in the full model changed from 0.101 (complete case analysis) to 0.088 (multiple imputation), a reduction of 12.9%.

### Discussion

While the regression coefficient for the interaction between DBDS and employment status was not statistically significant, the upper bound of the 95% CI did not exceed the null value. Thus, we cannot dismiss the possibility of effect modification outright (see “Limitations” below). If effect modification were to exist, then the inverse relation between behaviour and HRQoL would be stronger for retired versus employed caregivers. Based on the full regression model, the effect of DBDS on health utility score is − 0.0033 for retired caregivers (i.e., − 0.0003 + [− 0.003 × 1]) and − 0.0003 for employed caregivers (i.e., − 0.0003 + [− 0.003 × 0]). Since retired caregivers are probably at home more often than employed caregivers, and also less likely to be able to afford paid services such as respite care, retired caregivers have a greater chance of being exposed to difficult PwAD behaviours. Additionally, retired caregivers might experience more health challenges of their own compared to employed caregivers, simply because they are older. This can compound the experience of dealing with difficult behaviours and exacerbate HRQoL deficits.

The findings suggest a moderate effect size for retired caregivers. Recent work on the EQ-5D-5L [19], which has two additional response options per dimension compared to the -3L, reported a change in health utility score of 0.037 to be clinically important [20]. The DBDS score for a retired caregiver would have to increase by 11.22 points to produce a reduction in health utility score of 0.037 points, assuming effect modification by employment status (− 0.037 = − 0.0033 × 11.22). This degree of change is possible for retired caregivers, as evidenced by the interquartile range of DBDS scores, which exceeded 11.22 points. For employed caregivers, the effect is negligible because a 124-point increase in DBDS score would be required to reduce the health utility score by 0.037 points (− 0.0372 = − 0.0003 × 124). However, the maximum DBDS score is 112.

### Conclusion

No previous study has examined the effect of employment status on PwAD behaviours and informal caregiver HRQoL. The inverse association between these behaviours and caregiver HRQoL largely exists among retired caregivers. This novel finding suggests the need for policy makers to consider programs directed specifically at retired caregivers to provide relief from the burden of caregiving, e.g., tax deductions to reduce taxable income by the amount of paid respite care.

## Limitations

Many of the regression coefficients were not statistically significant, although the upper bounds of the 95% CIs either touched or slightly exceeded the null value of 0. This suggests the study was underpowered to detect changes in health utility scores. The study was also cross-sectional, meaning we could not assume changes in health utility scores would follow changes in PwAD behaviours. Future research should examine this topic longitudinally in larger samples. The study sample over-represented highly educated, high income caregivers, which suggests caution when applying the results to all caregivers.

## Abbreviations

AD: Alzheimer’s disease
CI: confidence interval
DBDS: Dementia Behaviour Disturbance Scale
HRQoL: health-related quality-of-life
PwAD: persons with Alzheimer’s disease

## References

[1] Richardson TJ, Lee SJ, Berg-Weger M, Grossberg GT (2013). Caregiver health: health of caregivers of Alzheimer’s and other dementia patients. Curr Psychiatry Rep.

[2] Park J (2016). Mortality from Alzheimer’s disease in Canada: a multiple-cause-of-death analysis, 2004 to 2011. Health Rep.

[3] Buckley JS, Salpeter SR (2015). A risk-benefit assessment of dementia medications: systematic review of the evidence. Drugs Aging.

[4] Brodaty H, Connors MH, Xu J, Woodward M, Ames D, PRIME Study Group (2015). The course of neuropsychiatric symptoms in dementia: a 3-year longitudinal study. J Am Med Dir Assoc.

[5] Zhao Q-F, Tan L, Wang H-F, Jiang T, Tan M-S, Tan L (2016). The prevalence of neuropsychiatric symptoms in Alzheimer’s disease: systematic review and meta-analysis. J Affect Disord.

[6] Fonareva I, Oken BS (2014). Physiological and functional consequences of caregiving for relatives with dementia. Int Psychogeriatr.

[7] van der Lee J, Bakker TJEM, Duivenvoorden HJ, Dröes R-M (2014). Multivariate models of subjective caregiver burden in dementia: a systematic review. Ageing Res Rev.

[8] Robert PH, Verhey FRJ, Byrne EJ, Hurt C, De Deyn PP, Nobili F (2005). Grouping for behavioral and psychological symptoms in dementia: clinical and biological aspects. Consensus paper of the European Alzheimer Disease Consortium. Eur Psychiatry J.

[9] García-Alberca JM, Cruz B, Lara JP, Garrido V, Lara A, Gris E (2012). Anxiety and depression are associated with coping strategies in caregivers of Alzheimer’s disease patients: results from the MÁLAGA-AD study. Int Psychogeriatr.

[10] Serrano-Aguilar PG, Lopez-Bastida J, Yanes-Lopez V (2006). Impact on health-related quality of life and perceived burden of informal caregivers of individuals with Alzheimer’s disease. Neuroepidemiology.

[11] Abdollahpour I, Nedjat S, Salimi Y, Noroozian M, Majdzadeh R (2015). Which variable is the strongest adjusted predictor of quality of life in caregivers of patients with dementia? QOL predictors in dementia caregivers. Psychogeriatrics.

[12] Zarit SH, Orr N, Zarit JM (1985). The hidden victims of Alzheimer’s disease: families under stress.

[13] Covinsky KE, Newcomer R, Fox P, Wood J, Sands L, Dane K (2003). Patient and caregiver characteristics associated with depression in caregivers of patients with dementia. J Gen Intern Med.

[14] Oremus M, Tarride J-E, Pullenayegum E, Clayton N, Mugford G, Godwin M (2015). Caregivers’ willingness-to-pay for Alzheimer’s disease medications in Canada. Dementia.

[15] Baumgarten M, Becker R, Gauthier S (1990). Validity and reliability of the dementia behavior disturbance scale. J Am Geriatr Soc.

[16] EuroQol Group (1990). EuroQol—a new facility for the measurement of health-related quality of life. Health Policy.

[17] Bansback N, Tsuchiya A, Brazier J, Anis A (2012). Canadian valuation of EQ-5D health states: preliminary value set and considerations for future valuation studies. PLoS ONE.

[18] Dieckmann L, Zarit SH, Zarit JM, Gatz M (1988). The Alzheimer’s disease knowledge test. Gerontologist.

[19] Herdman M, Gudex C, Lloyd A, Janssen M, Kind P, Parkin D (2011). Development and preliminary testing of the new five-level version of EQ-5D (EQ-5D-5L). Qual Life Res.

[20] McClure NS, Sayah FA, Xie F, Luo N, Johnson JA (2017). Instrument-defined estimates of the minimally important difference for EQ-5D-5L index scores. Value Health.

